# Dietary Supplementation With *Laminaria japonica* Extract Modulates Microbial Metabolic Functions, Improving Growth Performance, Innate Immunity, and Antioxidant Capacity in Juvenile *Procambarus clarkii* (GIRARD, 1852)

**DOI:** 10.1155/anu/6896135

**Published:** 2025-10-23

**Authors:** Minglang Cai, Weiqing Zhou, Xixun Zhou, Aimin Wang, Junzhi Zhang, Yi Hu

**Affiliations:** ^1^Fisheries College, Hunan Agricultural University, Changsha 410128, China; ^2^Yueyang Yumeikang Biotechnology Co., Ltd., Yueyang 414100, China; ^3^College of Marine and Biology Engineering, Yancheng Institute of Technology, Yancheng 224051, China

**Keywords:** antioxidant capacity, growth performance, gut microbiota, immune enzyme activity, *Laminaria japonica* extract, *Procambarus clarkii*

## Abstract

Concerns regarding food-borne interventions in crayfish have been raised due to excessive farming densities and the overuse of drugs in aquaculture. This research focused on examining the dose–response relationship of *Laminaria japonica* extract supplementation on growth performance, hepatopancreas antioxidant status, and innate immune function in crayfish, while exploring the microbiota-mediated metabolic pathways involved. A total of 750 juvenile crayfish (4.00 g) were randomly assigned to five treatments and fed diets supplemented with *L. japonica* extract at concentrations of 0, 500, 1000, 1500, and 2000 mg/kg for 42 days. The results demonstrated that dietary *L. japonica* extract improved the growth and hepatic health status, as indicated by well-structured hepatic tubules and increased fibroblast cells, as well as lower hemolymph glutamic oxaloacetic transaminase (GOT) level (*p* < 0.05). Dietary supplementation with 1500 mg/kg *L. japonica* extracts significantly increased hemolymph lysozyme (LZM) and acid phosphatase (ACP) activities (*p* < 0.05). Additionally, *L. japonica* extract supplementation considerably increased hepatopancreas glutathione (GSH) content and activities of catalase (CAT), superoxide dismutase (SOD), and GSH reductase (GR) (*p* < 0.05). Furthermore, dietary *L. japonica* extract alleviated microbial dysbiosis, as characterized by the observed decrease in opportunistic pathogens *Citrobacter* and *Vibrio* and an increase in beneficial taxa *Tyzzerella*. Further findings found that 349 differential microbes were identified, with *Bacillus*, *Chryseobacterium*, and *Prevotella* playing key roles. In summary, the optimal dietary inclusion level of *L. japonica* extract was recommended to be 1507.89–1614.26 mg/kg. Dietary supplementation with 1500 mg/kg of *L. japonica* extract improved the immunity and antioxidant capacities of crayfish by reshaping microbial co-occurrence networks.

## 1. Introduction

*Procambarus clarkii*, commonly known as crayfish, is an important aquatic crustacean in China [1]. With moderate protein content, flavorful meat, and rich amino acids, crayfish are highly popular among consumers [2]. In recent years, the advancement of aquaculture techniques, improvements in the industrial chain, and the rapid development of cold chain distribution and logistics have contributed to sustained growth in both aquaculture areas and industry scale. Specifically, the total crayfish production in China was 3,161,000 tons in 2023, reflecting a 9.35% year-on-year increase compared to 2022 (2,890,700 tons) [3]. Although the output of the crayfish industry has exceeded CNY400 billion, several key challenges are hindering its healthy and sustainable development, exacerbated by the rapid expansion of crayfish farming. These challenges include an increase in the proportion of poor-quality crayfish, such as iron-shell and low-specification varieties [4, 5], due to changes in light and nutritional factors, which negatively impact the taste of crayfish. Furthermore, inadequate nutritional research and irrational diet formulations have led to suboptimal growth performance [6] and high mortality rates among larvae during metamorphosis and development [7]. Additionally, excessive stocking densities contribute to the frequent outbreak of diseases [8, 9], leading to the widespread use of antibiotics and other drugs, which, in turn, compromise food safety. To address these issues, functional feeds specifically tailored for crayfish offer considerable potential in improving aquaculture sustainability and food safety.

Kelp (*Laminaria japonica*) polysaccharides are natural active substances mainly found in *L. japonica*, with complex and diverse structures and uneven molecular weights [10–12]. With a good safety profile, macro algae, including *L. japonica*, have often been reported for their application in food [10, 13–15], encouraging their use as a feed additive. Previous studies have demonstrated that dietary supplementation with *L. japonica* extract enhances growth [16, 17], lipid metabolism [18, 19], and physiological activity [20] in animals. Earlier reports revealed that *L. japonica* had a good protective effect on carbon tetrachloride-induced acute liver injury, which could reduce the serum alanine aminotransferase and aspartate aminotransferase viability [21, 22], coupled with the increased superoxide dismutase (SOD) activity. However, *L. japonica* carries a higher proportion of nonstarch polysaccharides, with its excessive use highlighting its antinutritional effects [23]. The intestinal tract serves as a crucial interface for polysaccharide bioactivity, maintaining a complex microbial ecosystem while acting as a primary barrier against pathogenic microorganisms [24, 25]. This vital organ enhances host defense mechanisms through the production of immunologically active compounds and the regulation of microbial homeostasis [26]. It was reported that beneficial bacteria increased in the digestive tract of animals subjected to fucoidan-rich *L. japonica*, leading to microbial dysbiosis [27–29]. Emerging evidence has established a strong association between intestinal microbial imbalance and inflammatory responses [30–32], mediated through modulation of gene expression patterns and tissue structural alterations [33–35]. The complexity of this relationship stems from intricate intermicrobial networks and their inherent structural properties, which collectively influence microbial homeostasis [36, 37]. Currently, fewer studies have been reported on the application of *L. japonica* polysaccharides in the study of aquatic microorganisms and their specific mechanism.

Building upon the common challenges in the development of the crayfish industry, the current investigation explores the potential of dietary *L. japonica* extract supplementation at varying concentrations to enhance crustacean health status, focusing on survival capacity, immune competence, and growth parameters. Consequently, elucidating the mechanisms through which intestinal microbiota sustain host homeostasis necessitates a comprehensive investigation into both the adaptive responses to dietary *L. japonica* supplementation and the functional contributions of key microbes. This study provided a food-borne approach that strongly supported the healthy and sustainable development of the crayfish industry in China.

## 2. Methods and Materials

### 2.1. Experimental Diets

The experimental diets were formulated based on the commercial feed composition for *P. clarkii*. Feed preparation involved initial ingredient processing at Hunan Agricultural University (HUNAU), including particle size reduction and preliminary mixing, followed by final granulation processing at Yuehai Feed Co. (Guangdong, China). The basal diet (CK), containing 33.10% crude protein and 6.2% crude fat, was used to domesticate *P. clarkii*. Four graded levels of *L. japonica* extract (500, 1000, 1500, and 2000 mg/kg) were incorporated into the control formulation to formulate experimental diets, labeled as LJP0.5, LJP1.0, LJP1.5, and LJP2.0, corresponding to their respective supplementation concentrations. Notably, the *L. japonica* extract was prepared by hot water extraction followed by ethanol precipitation, and spray-drying to obtain a powdered form. The proximate composition of *L. japonica* extract is presented in the Supporting Information 1. The feed ingredients and the proximate nutrient levels are listed in Table 1.

For preparation, the raw materials were passed through a 60-mesh sieve, after which they were premixed in small quantities. The homogenized powder formulation was subsequently blended with soybean oil and processed into uniform pellets (2 mm diameter) using a laboratory extruder. The prepared feed was subjected to air-drying until achieving optimal moisture levels (<10%) before final storage at −20°C for experimental use. Nutritional composition analysis of experimental diets was performed according to standardized protocols [38], with protein content quantified through the Kjeldahl nitrogen determination method (GB/T 6432-1994) and crude fat content assessed via Soxhlet extraction methodology (GB/T 6433-2006), in compliance with Chinese national analytical standards.

### 2.2. Feeding Management

The feeding trial was conducted at the Aquatic Biotechnology Research Center in Yiyang, Hunan Province, utilizing randomly sex-distributed crayfish populations. Prior to experimental initiation, crayfish underwent a 48-h fasting period followed by a 14-day acclimatization phase, during which they were maintained on the control diet to ensure environmental adaptation and physiological stabilization. During the acclimatization phase, a total of 750 juvenile crayfish, each weighing ~4.00 g, were randomly allocated to 15 net cages (2.0 × 2.0 × 1.5 m) at a stocking density of 50 individuals per unit. The feeding regimen consisted of twice-daily rations (07:00–08:00 and 17:00–18:00) equivalent to 8% of total crayfish weight, with weekly adjustments based on growth performance during the 42-day trial. Environmental parameters were systematically monitored and regulated to maintain optimal aquatic conditions for crustacean development.

### 2.3. Growth Performance Estimation

Morphometric measurements, including body length and weight, were recorded for all crayfish within each cage. Growth performance indices were estimated using established biometric equations [38]. The formulae for all growth and morphometric parameters are listed below:

  $$\begin{array}{c} \text{Weight gain rate }\left(\text{WGR},\%\right)=\left[\left(\text{Final weight}-\text{initial weight}\right)/\text{initial weight}\right]\times 100, \end{array}$$

  $$\begin{array}{c} \text{Specific growth rate }\left(\text{SGR},\%/\text{day}\right)=\left[\left(\text{Ln}\left(\text{final weight}\right)-\text{Ln}\left(\text{initial weight}\right)\right)/\text{days}\right]\times 100, \end{array}$$

  $$\begin{array}{c} \text{Feed conversion ratio }\left(\text{FCR}\right)=\text{Total feed intake}/\text{total weight gain}, \end{array}$$

  $$\begin{array}{c} \text{Hepatosomatic index }\left(\text{HSI},\%\right)=\left(\text{Hepatopancreas weight}/\text{body weight}\right)\times 100, \end{array}$$

  $$\begin{array}{c} \text{Condition factor }\left(\text{CF},g/{\text{cm}}^{3}\right)=\left[\text{Body weight}/{\left(\text{body length}\right)}^{3}\right]\times 100. \end{array}$$

### 2.4. Determination of Malondialdehyde (MDA) Content and Enzyme Activity

For biochemical analyses, hemolymph was obtained from the pericardial sinus using a sterile syringe, which was collected from 12 representative individuals per treatment group (four individuals per net cage). Following collection, samples were aliquoted, maintained at 4°C, and subsequently centrifuged (4000 × *g*, 10 min) to obtain supernatant, which was stored at −80°C. Parallel sampling was conducted for hepatopancreas collection from separate individuals (six individuals per treatment group), with all tissue samples flash-frozen in liquid nitrogen and archived at −80°C for subsequent processing. Oxidative stress markers and enzymatic activities were evaluated using hemolymph and hepatopancreatic tissue samples. MDA (A003-1) concentration was quantified in both sample types, while hemolymph was specifically analyzed for glutamic oxaloacetic transaminase (GOT, C010-2-1), acid phosphatase (ACP, A060-2-2), and lysozyme (LZM, A050-1-1) activities. Hepatopancreatic samples were processed to determine total protein (TP, A045-4-2) content, glutathione (GSH, A006-2-1) levels, and the activities of key antioxidant enzymes, including SOD (A001-1-1), catalase (CAT, A007-1-1), GSH S-transferase (GST, A004-1-1), GSH reductase (GR, A062-1-1), and GSH peroxidase (GPx, A005-1-2). Commercial assay kits (Nanjing Jiancheng Bioengineering Institute) were utilized following standardized protocols provided by the manufacturer.

### 2.5. Histological Analysis and Image Processing

Parallel sampling with hemolymph samples was conducted for hepatopancreas collection from separate individuals (six individuals per treatment group), with three hepatopancreas samples selected for tissue sections preparation. Hepatopancreatic tissue sections were processed using standard hematoxylin–eosin (H&E) staining protocols (see Supporting Information 3 for detailed methodology). Digital image analysis was performed on 8-bit converted histological images using ImageJ software (NIH, USA) for quantitative morphological assessment. The hepatic tubules of the hepatopancreas were graphically fitted and measured using a modified threshold (Threshold: 0 and 141, Color: Red, Model: Default). The H&E-stained hepatopancreas slices were enhanced and colorized in gray and red. Subsequently, F cells in the hepatic tubules were fitted, identified, and counted based on a size range of 12-to-30-pixel^2^ using the function “analyze particles.”

### 2.6. Gene Expression Detection

Parallel sampling with hemolymph samples was conducted for hepatopancreas collection from separate individuals (six individuals per treatment group), with all hepatopancreas samples flash-frozen in liquid nitrogen and archived at −80°C for subsequent processing. Total RNA isolation from hepatopancreas samples, including quality assessment and cDNA synthesis, was conducted as described in Supporting Information 4. β-actin served as the endogenous control based on established crustacean sequences from NCBI databases. Specific primers targeting antioxidant pathway genes (*nrf2*, *gr*, *mmnsod*, *gst*, *gpx*, and *cat*) and immune response-related genes (*nfkb*, *tlr*, *lzm*, and *alf*) were designed using microchat and commercially synthesized (Tsingke Biotechnology, Shanghai). The real-time PCR procedure followed the methodology outlined in a previous study [39]. Following validation of primer amplification efficiency (90%–110%), quantitative real-time PCR analysis was performed using the comparative Ct method (2^−ΔΔCt^) for relative gene expression quantification. Primer sequences and corresponding details are presented in Table 2.

### 2.7. Amplicon Sequencing and Microbial Community Profiling

Parallel sampling with hemolymph samples was conducted for intestinal content collection from separate individuals (six individuals per treatment group), with all intestinal content samples flash-frozen in liquid nitrogen and archived at −80°C for subsequent processing. Intestinal microbiota analysis was performed through 16S rRNA gene sequencing of 30 intestinal content samples using Illumina paired-end sequencing technology (Personal Biotechnology Co., Shanghai). Sequence data preprocessing, including quality filtering and annotation procedures, is detailed in Supporting Information 5. Unique barcode sequences (6 bp) were incorporated into forward primers during synthesis to facilitate sample multiplexing. Raw sequencing data have been deposited in the Sequence Read Archive (SRA) under accession number PRJNA854610.

To enhance analytical precision, rare amplicon sequence variants (ASVs) were filtered out prior to taxonomic classification and abundance estimation. ASV-based operational taxonomic units (OTUs) were employed for subsequent microbial community analysis. Beta diversity patterns were examined through principal co-ordinate analysis (PCoA) with graphical representation using the ggplot2 package. Differential abundance analysis was conducted using the Limma package, implementing a significance threshold of *p* < 0.05 and a minimum fold change of 1 for biomarker identification. Microbial co-occurrence networks were constructed from five adjacency matrices, excluding OTUs with prevalence below two samples, using Spearman correlation coefficients. Network topology was determined through random matrix theory (RMT) analysis with a standardized similarity cutoff of 0.93. Nonstochastic co-occurrence patterns were observed in the microbial networks, as indicated by the comparison of empirical and random networks across various network characteristics (Table 3).

### 2.8. General Statistical Analyses

Statistical evaluations were performed using an interactive web-based platform (https://mineraltsai.shinyapps.io/shinymicrochat). Parametric data meeting assumptions of variance homogeneity and normal distribution were analyzed through one-way ANOVA with Tukey's post hoc tests for multiple comparisons. Nonparametric datasets were subjected to Kruskal–Wallis analysis followed by Dunn's test for pairwise comparisons. Statistical significance was determined at *p*  < 0.05, with corresponding indicators presented in all tabular data. Besides, the linear and quadratic responses to dietary inclusion levels of *L. japonica* extract on the crayfish phenotypes were analyzed using orthogonal polynomial contrast (OPC). Although the theoretical effect model for dietary *L. japonica* extracts inclusion levels includes up to level *n* – 1 ([number of groups] – 1), for clarity and esthetic purposes, at most 2nd-level responses were shown in the tables and figures, that is, linear, quadratic.

## 3. Results

### 3.1. Growth and Morphology-Related Parameters

**Figure 1:**
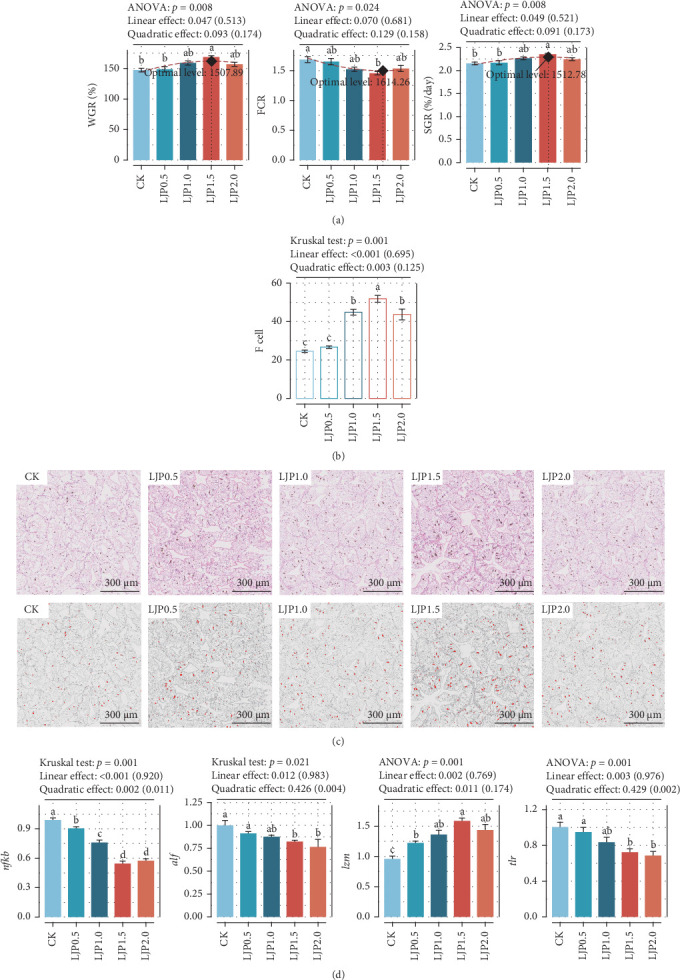
Growth, feed utilization, hepatic histological morphology observation, and immune-related gene expression levels. (A) Quadratic-curve fitting models for growth and feed utilization. WGR, weight gain rate. SGR, specific growth rate. FCR, feed conversion rate. (B) Bar plot of F cells in hepatic tubules. The presented data represent the mean of six replicates (mean ± SEM). Different letters indicate statistical differences at a significance level of *p* < 0.05. (C) Hepatopancreas histological morphology originated from hepatopancreas slices stained by hematoxylin–eosin (H&E). Magnification: × 100. (D) Immune response-related gene expression levels. *nfkb*: nuclear factor kappa B. *lzm*: lysozyme. *alf*: antilipopolysaccharide factor. *tlr*: Toll-like receptor. The presented data represent the mean of six replicates (mean ± SEM). Different letters indicate statistical differences at a significance level of *p*  < 0.05. Orthogonal polynomial contrasts were used to evaluate the impact of dietary *L. japonica* extract level on each parameter. The values listed in the brackets were the fitted value (explained degree) of the corresponding response models, and the character string outside the brackets denotes the *p*-value.

As presented in Table 4, the final average weight (FAW), weight gain rate (WGR), specific growth rate (SGR), feed conversion ratio (FCR), and hepatosomatic index (HSI) in crayfish were linearly altered by dietary *L. japonica* extract supplementation (*p* < 0.05), while no significant difference could be seen in the survival rate of crayfish (*p* > 0.05). Specifically, dietary *L. japonica* extract supplementation significantly increased FAW, WGR, and SGR (*p* < 0.05), with the highest values observed at a supplementation level of 1500 mg/kg. Conversely, FCR decreased and then increased as dietary *L. japonica* extract supplementation increased, while the opposite trend was observed for condition factor (CF) (*p* > 0.05). Based on the quadratic-effect model, the optimal inclusion level of *L. japonica* extract in crayfish feed was estimated to be between 1507.89 and 1614.26 mg/kg, as determined by the WGR, SGR, and FCR (Figure 1A).

### 3.2. Hemolymph Biochemical Indices and Hepatopancreas Morphology

As indicated in Table 5, hemolymph MDA and GOT levels linearly decreased in crayfish given *L. japonica* extract-supplemented feed compared to those of the CK group, while increasing the activities of LZM and ACP (*p* < 0.05). Notably, dietary *L. japonica* extract supplementation at 1500 mg/kg considerably lowered the hemolymph MDA content compared to the other *L. japonica* extract-supplemented groups (*p* < 0.05), together with the increased ACP and LZM activities (*p* < 0.05).

According to Figure 1C, crayfish-fed diets supplemented with *L. japonica* extract exhibited well-organized hepatopancreatic structures, characterized by regular cell morphology, distinct polygonal lumens, and tightly connected junctions between hepatic tubules. In contrast, the hepatopancreas of crayfish in the CK group showed loosely joined hepatic tubules without wrinkles, along with ruptured basement membranes of hepatopancreatic cells and a reduced number of F cells, compared to crayfish fed *L. japonica* extract at concentrations of 1000–2000 mg/kg (Figure 1B).

### 3.3. Hepatopancreas Inflammation-Related mRNA Expression Patterns

As revealed in Figure 1D, dietary *L. japonica* extract feeding considerably downregulated *nfkb*, *alf*, and *tlr* compared to the CK treatment (*p* < 0.05), coupled with the increased *lzm* mRNA expression level (*p* < 0.05). Notably, a high supplementation with *L. japonica* extract at 1500–2000 mg/kg contributed to the increased mRNA expression levels of *lzm* than that of the low *L. japonica* extract-supplemented groups.

### 3.4. Hepatopancreas MDA Content and *nrf2* Signaling Pathway

**Figure 2:**
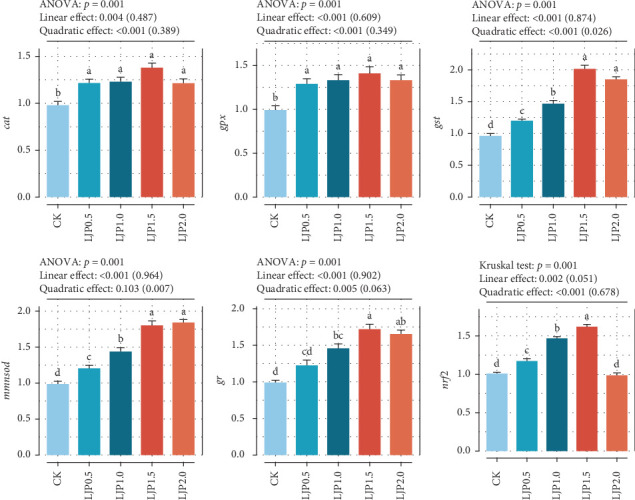
Hepatopancreas antioxidant-related gene expression levels. Note: *cat*, catalase. *gpx*: glutathione peroxidase. *gst*: glutathione s-transferase. *mmnsod*, mitochondrial manganese superoxide dismutase. *nrf2*: NF-E2-related nuclear factor. *gr*: glutathione reductase. The presented data represent the mean of six replicates (mean ± SEM). Different letters indicate statistical differences at a significance level of *p* < 0.05. Orthogonal polynomial contrasts were used to evaluate the impact of dietary *L. japonica* extract level on each parameter. The values listed in the brackets were the fitted value (explained degree) of the corresponding response models, and the character string outside the brackets denotes the *p*-value.

As presented in Table 6, dietary *L. japonica* extract supplementation considerably decreased MDA accumulation while enhancing GSH levels and the activities of CAT, SOD, GST, GR, and GPx (*p* < 0.05). Similarly, crayfish given *L. japonica* extract supplementation at 500–1500 mg/kg exhibited higher *nrf2*, *cat*, *gr*, *gst*, and *gpx* mRNA expression levels than those of the CK group (*p* < 0.05, Figure 2). Gene expression profiling demonstrated upregulation of *nrf2*, *cat*, *gr*, *gst*, and *gpx* in crayfish given 500–1500 mg/kg *L. japonica* extract compared to the CK treatment group (*p* < 0.05, Figure 2).

### 3.5. Gut Microbiota Summary

**Figure 3:**
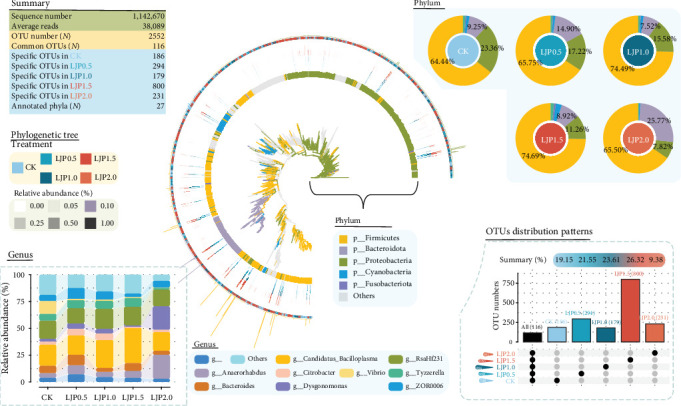
Microbial community structure in the gut of *P. clarkia*. Note: Barcharts of relative abundance at the phylum level explained the distribution of dominant taxa. The upset plot explained the number of unique and shared microbes in each group. The histogram of relative abundance at the genus level explained the distribution of dominant taxa. A phylogenetic tree was drawn using ggtree. The inner to outer frontal layout is microbial evolutionary branches (colored by different phyla), microbial rings (colored by different phyla), and relative abundance heatmaps (colored by group).

**Figure 4:**
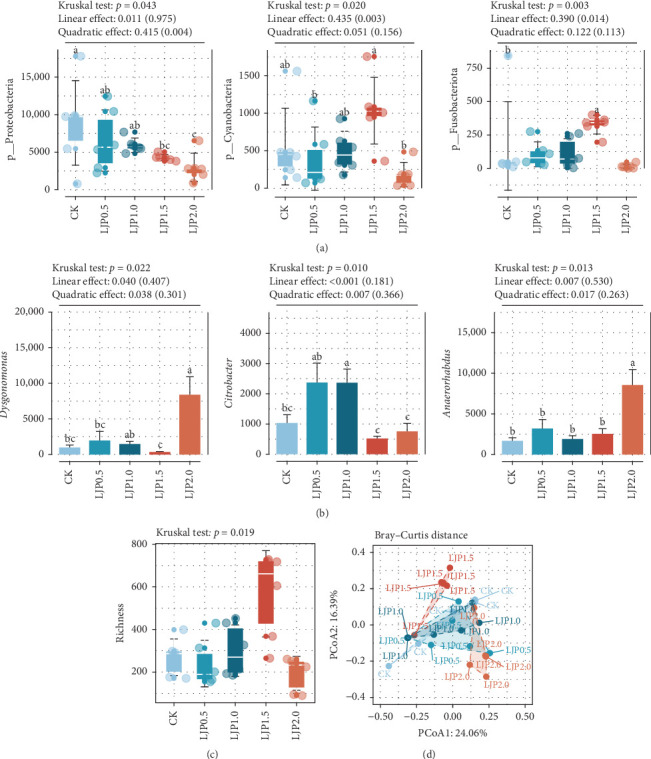
Key taxa, alpha, and beta diversity of microbial community in the gut of *P. clarkia*. (A, B) Significant changes in dominant phyla and genera. (C) Boxplot of the richness index. (D) Beta diversity using PCoA analysis based on Bray–Curtis distance. “*⁣*^*∗*^” indicates statistically significant differences at a significance level of *p* < 0.05.

Microbial community analysis was conducted through 16S rRNA gene sequencing of intestinal samples from all experimental groups (*n* = 30). After quality filtering and removal of low-prevalence OTUs (present in less than two samples), a total of 1,142,670 high-quality sequences were obtained, yielding an average of 38,089 reads per sample with more than 99% coverage. Taxonomic classification identified 2552 bacterial OTUs across all groups, with treatment-specific distributions as follows: CK (798 OTUs, 19.15%), LJP0.5 (898 OTUs, 21.55%), LJP1.0 (984 OTUs, 23.61%), LJP1.5 (1097 OTUs, 26.32%), and LJP2.0 (391 OTUs, 9.38%) (Figure 3). Notably, 116 microbes were presented across all treatments, and the numbers of OTUs unique to each treatment were 186 (CK), 294 (LJP0.5), 179 (LJP1.0), 800 (LJP1.5), and 231 (LJP2.0), respectively. The richness index showed considerable differences among the CK and *L. japonica* extract-supplemented groups (*p* < 0.05, Figure 4C), with the LJP1.5 group demonstrating superior diversity indices compared to other dietary regimens. PCoA based on Bray–Curtis dissimilarity (PCoA1: 24.06%, PCoA2: 16.39%) effectively captured community-level shifts in intestinal microbiota composition across different supplementation levels (Figure 4D). Distinct clustering patterns were observed, with LJP1.5 and LJP2.0 groups forming separate clusters from lower supplementation groups (CK, LJP0.5, LJP1.0). These group-specific microbial profiles were statistically validated through multiple nonparametric permutation tests (Supporting Information 6).

### 3.6. Key Taxa Alterations

Microbial sequence profiling analysis identified 27 phyla, with Firmicutes, Bacteroidota, Proteobacteria, Cyanobacteria, and Fusobacteriota recognized as the predominant phyla (Figure 3). Dietary *L. japonica* extract supplementation led to considerable differences in Proteobacteria, Cyanobacteria, and Fusobacteriota (*p* < 0.05, Figure 4A), with a marked decrease in Proteobacteria observed in *L. japonica* extract-treated groups. The crayfish of LJP1.5 food-borne supplementation treatment exhibited maximal representation of Cyanobacteria and Fusobacteriota. At the genus level, the microbiota was characterized by the prevalence of Firmicutes-associated taxa (*Candidatus* Bacilloplasma, *RsaHf231*, *Anaerorhabdus*, *Tyzzerella*), Proteobacteria (*Citrobacter*, *Vibrio*), and Bacteroidota (*Bacteroides*, *Dysgonomonas*). Dietary *L. japonica* extract supplementation led to considerable alteration in the abundance of *Anaerorhabdus*, *Dysgonomonas*, and *Citrobacter* (*p* < 0.05, Figure 4B), and decreased *Vibrio* (*p* > 0.05). Specifically, high *L. japonica* extract supplementation at 1500 and 2000 mg/kg significantly decreased the relative abundance of *Citrobacter*, whereas *L. japonica* extract at 2000 mg/kg considerably increased the *Anaerorhabdus* and *Dysgonomonas* (*p* < 0.05). Furthermore, a higher abundance of *Tyzzerella* was observed in crayfish given diets supplemented with *L. japonica* extract at 500–1500 mg/kg than that of the *L. japonica* extract-free group.

### 3.7. Microbial Differential Analysis

**Figure 5:**
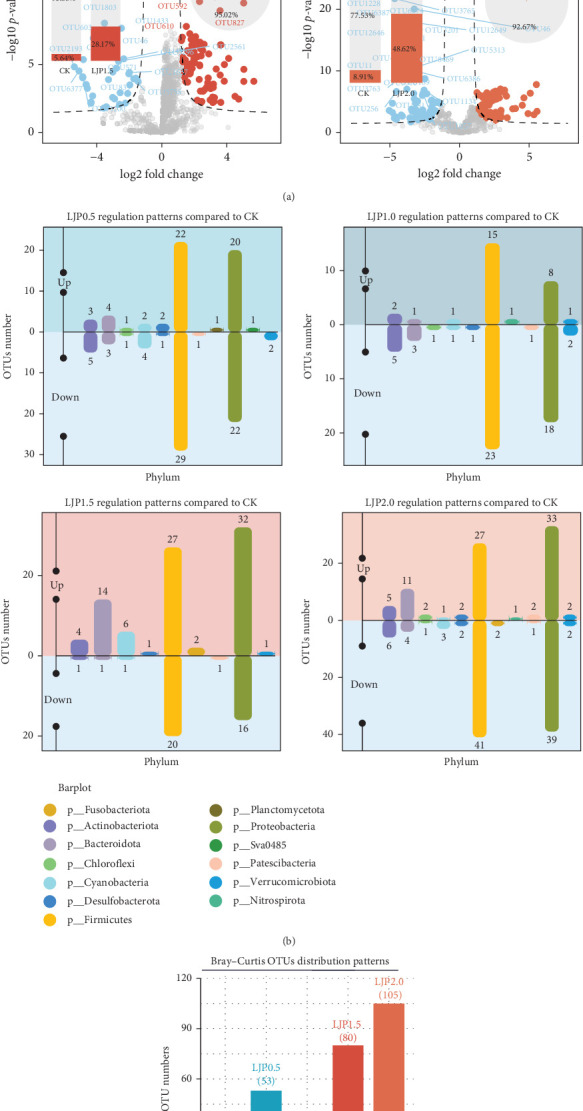
Microbial community differential analysis in the gut of *P. clarkia*. (A) The differential OTUs in the *L. japonica* extract-supplemented groups compared to the CK group. (B) Distribution of differential OTUs at the phyla level. (C) Distribution of differential OTUs at the species level.

It was further revealed that the gut bacterial profiles of crayfish given *L. japonica* extract-supplemented diets reported 56 (LJP0.5), 29 (LJP1.0), 87 (LJP1.5), and 86 (LJP2.0) upregulated OTUs and 68 (LJP0.5), 55 (LJP1.0), 40 (LJP1.5), and 101 (LJP2.0) down-regulated OTUs compared to the CK group (Figure 5A). The gut bacterial profiles of crayfish-fed diets supplemented with *L. japonica extract* showed significant differences, particularly within the Firmicutes and Proteobacteria phyla (Figure 5B). Additionally, a total of 349 differential OTUs were identified in the *L. japonica* extract-supplemented groups compared to the CK group (Figure 5C), with 87 OTUs being notably influenced by the dietary inclusion of *L. japonica* extract.

### 3.8. Microbial Co-Occurrence Network Profiling

**Figure 6:**
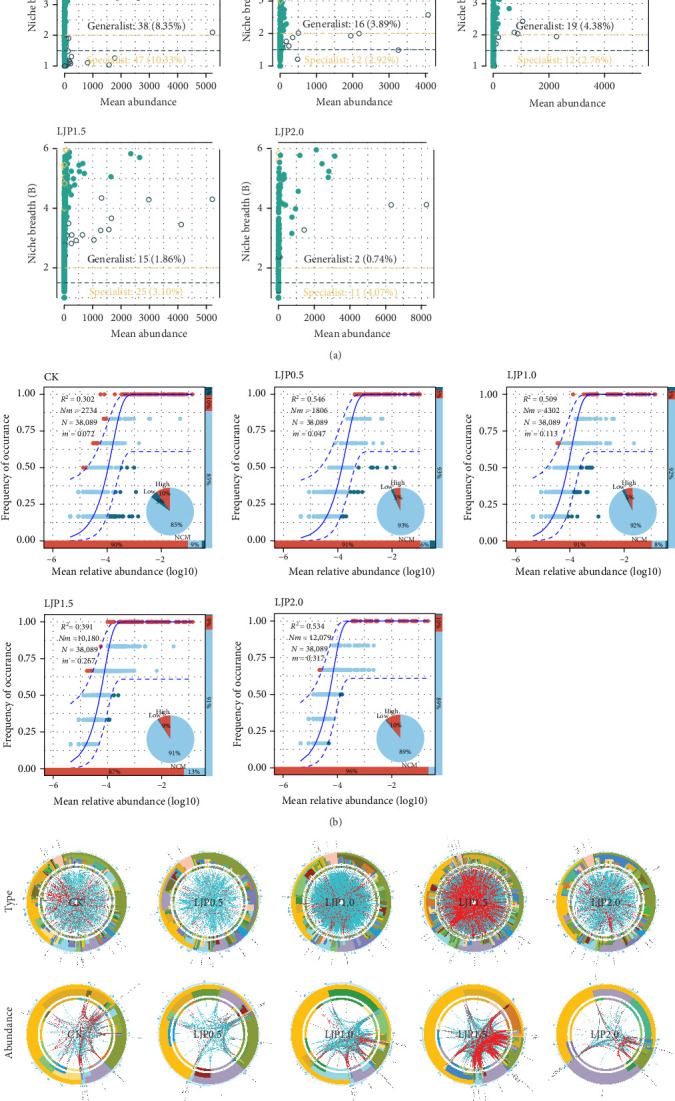
Molecular ecological network analysis in the gut microbiota of *P. clarkia*. (A) Niche breadth distribution of microbes and the differentiation of generalists and specialists. (B) Community assembly process evaluation based on a neutral community model. A higher *R*^2^ represents a better fit of the neutral model, which means a greater contribution of stochastic processes to the construction of the community. (C) Circos plot for visualizing microbial interactions. The first row is plotted based on microbial species, and the second row is plotted based on abundance. The bands are, from outside to inside, phylum, class, order, family, genus, and species. Blue edges represent positively correlated microbial interactions. Red edges represent positively correlated microbial interactions.

Microbial community assembly patterns were evaluated to determine the underlying ecological processes governing microbiota composition in response to dietary supplementation. Neutral model analysis revealed poor fitting for all treatment groups (Figure 6B), suggesting the predominance of deterministic processes in community assembly. Further, niche-based assessment identified both generalist and specialist taxa across all groups (Figure 6A), with the CK group displaying the highest abundance of these ecological strategists. With the *L. japonica* extract supplementation included in the feed being, the sum of generalists and specialists in the gut bacterial profiles in crayfish increased and then decreased, reaching the highest peak in the LJP1.5 group as well as the lowest in the LJP2.0, further implying that the dietary *L. japonica* extract supplementation could reshape the gut microbial ecological roles and weaken the bacterial profiles at a higher concentration of 2000 mg/kg.

Molecular ecological networks were constructed to elucidate microbiota-mediated regulatory mechanisms under varying *L. japonica* supplementation levels (Figure 6C). Comparative analysis revealed significantly lower *avg*CC, *M*, and *GD* in random networks versus empirical networks (Table 3). The LJP1.5 group exhibited the most complex network, characterized by maximal node connectivity and interspecies interactions. Network architecture analysis demonstrated strong modularity across all groups (*M* > 0.3), with module numbers ranging from 14 (LJP1.0) to 22 (LJP0.5, LJP1.5). Notably, positive correlations dominated microbial interactions (94.02%–99.26%), indicating a strong preference for co-operative relationships over competitive exclusion. The numbers of negative-linked edges varied, with the CK network possessing the highest proportion of negative correlation (5.98%), whereas dietary *L. japonica* extract supplementation at 1500 mg/kg enabled the most negative inter-species interactions than that of the gut bacterial profiles in crayfish given the *L. japonica* extract-supplemented diets.

**Figure 7:**
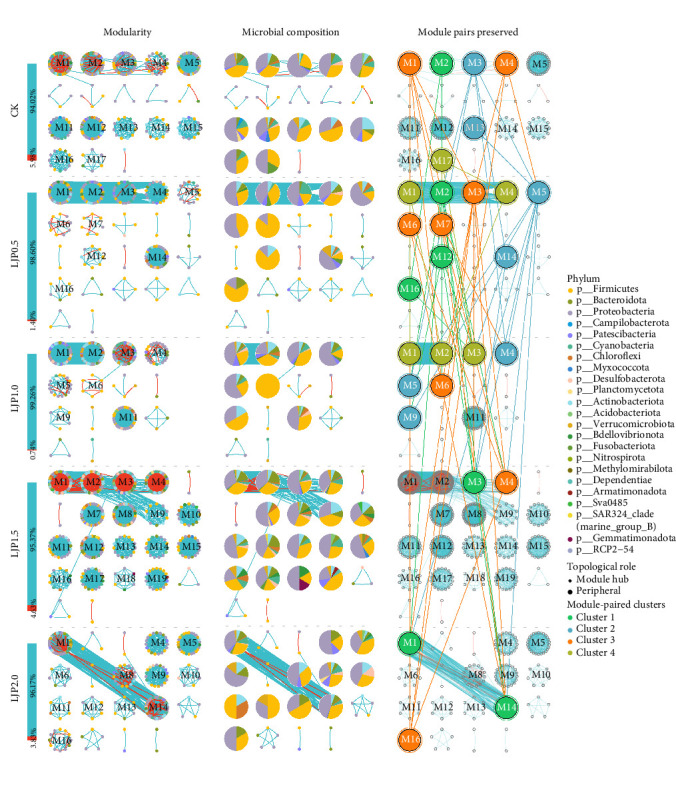
Modularity and ecological roles of molecular ecological network analysis. Note: Co-occurrence network plot for visualizing microbial interactions. Blue edges represent positively correlated microbial interactions. Red edges represent positively correlated microbial interactions. The 1st column is visualized for microbial interaction strengths. The 2nd column is visualized based on the modular differentiation properties of microbial networks. The 3rd column of the layer is the taxonomic composition of all the network modules. The 4th column of the layer is mining the dissimilarity of specific network modules. Extract specific modules of microbial networks and highlight the abundance composition at the phyla level. Moreover, modules represent assemblages of microbes that can perform specific ecological functions. The modules of microbial network from each group were analyzed for similarity of modules by Fisher's test, and modules were considered similar if the differences were found to be significant. Similar categories of modules were grouped into module clusters and colored accordingly. This allowed the retention of microbial network modules and the potential ecological functions that could be performed under different treatments to be explored.

Network topology profiling identified 1370 module pairs and 95 individual modules across all microbial co-occurrence networks. Differential analysis revealed 61 conserved module pairs and 28 single modules (4.45% of total) that clustered into four distinct functional groups (Figure 7). Both CK and LJP0.5 groups exhibited modules from all four clusters, while higher supplementation levels (1500–2000 mg/kg) were characterized by modules primarily from clusters 1 and 3. Cluster 3 modules were universally present across all groups, whereas cluster 1 was absent in the LJP1.0 group. Notably, cluster 2 modules were exclusively identified in CK and low-supplementation groups (LJP0.5, LJP1.0), as evidenced by tightly connected modules M3/M13 (CK), M5/M14 (LJP0.5), and M4/M5/M9 (LJP1.0).

### 3.9. Identification of Phenotype-Associated Microbial Biomarkers

**Figure 8:**
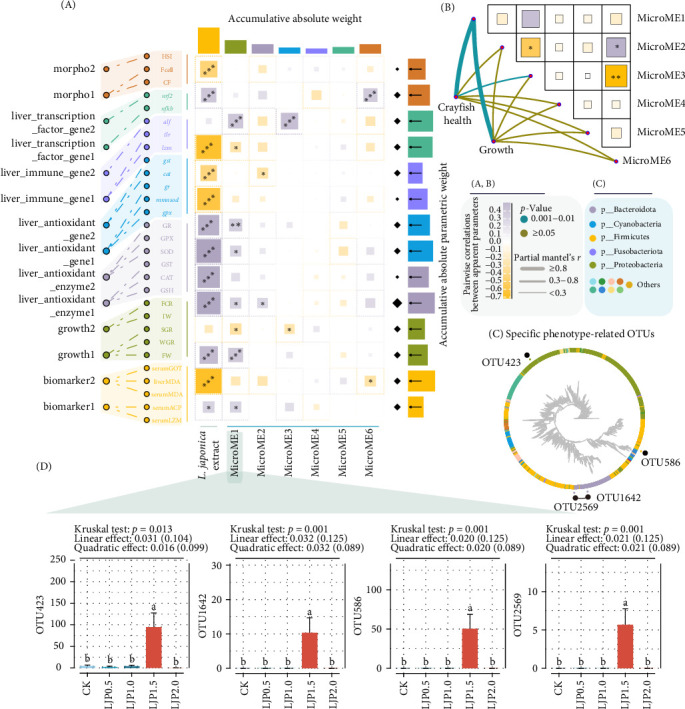
Potential regulatory patterns in *P. clarkii* subjected to varying dosages of dietary *L. japonica* extract supplementation. (A) Correlation analysis based on the Spearman coefficient was used for the interactions between the module eigengenes extracted from 349 differential OTUs abundance matrix and the secondary module eigenvectors extracted from the phenotype matrix. MicroMEs, microbial module eigengenes. (B) Mantel's test was used for the interactions between the phenotype matrix and the module eigengenes extracted from 349 differential OTUs abundance matrix. (C) The phylogenetic roles of biomarkers derived from microbial modular 1. (D) Boxplot of identified biomarkers. “*⁣*^*∗*^” indicates statistically significant differences at a significance level of *p* < 0.05.

Deep mining of microbial module eigenvectors (MicroMEs) identified six significant modules correlated with phenotypic variations, along with a dose–response module for *L. japonica* extract supplementation (Figure 8A, B). The matrices derived from the phenotypic data were categorized into 14 featured modules. Notably, MicroME1 exhibited a similar pattern as the *L. japonica* extract dose in regulating crayfish phenotypes. Through comprehensive module classification, host phenotypic responses were stratified into two distinct categories: growth-related (module 3) and health-related (modules 1, 5, 7, 8, 12) based on MicroME1 pairwise comparisons. Additionally, it was revealed MicroME1 as a principal functional module influencing core host phenotypes, demonstrated by significant correlations with “Crayfish health” and “Growth” (*r* > 0.3) (Figure 8B). Phenotype-associated biomarker analysis identified strong positive associations (*p* < 0.05, *r* > 0.4) between MicroME1 and key immunological indicators (ACP, LZM) within the phenotypic matrix (Figure 8A). This was also the case for growth1, liver_antioxidant_enzyme1, liver_antioxidant_gene1, liver_antioxidant_gene2, and liver_transcriptionfactor_gene2, as well as negatively correlated with growth2 and liver_transcriptionfactor_gene1. Integrative microphenotype analysis identified four key microbial taxa significantly associated with MicroME1: *Bacillus* (OTU586, assigned to Firmicutes), *Chryseobacterium* and *Prevotella* (OTU1642 & OTU2569, assigned to Bacteroidota), and *Nautella* (OTU423, assigned to Proteobacteria) (Figure 8C). Notably, dietary *L. japonica* extract supplementation at 1500 mg/kg significantly increased the abundance of all four major OTUs compared to other groups (*p* < 0.05, Figure 8D). Notably, the gut bacterial profiles of crayfish given 1500 mg/kg *L. japonica* extract showed significantly elevated abundance of these microbial biomarkers compared to other treatments (*p* < 0.05, Figure 8D).

## 4. Discussion

The growth of aquatic animals is a key economic factor in aquaculture, making it one of the most important traits for breeding and production. Dietary nutrients play a crucial role in the growth and maturation of aquatic animals [40, 41]. Of these, plant-derived ingredients are increasingly favored by aquafeed manufacturers and researchers due to their availability and beneficial biological activity [42–44]. Numerous studies have demonstrated the potential of *L. japonica* for inclusion in aquatic animal feeds, with positive effects on both growth and overall health [45, 46]. In the present study, moderate supplementation of *L. japonica* extract promoted the growth and CF of crayfish, although it resulted in a decrease in the HSI. It is suggested that the growth-promoting effect of *L. japonica* extract is likely attributable to the bioactive compounds, including proteins, polysaccharides, pigments, minerals, polyphenols, and polyunsaturated fatty acids that it contains [47]. In addition, kelp constitutes betaine and other trace components with food-attracting properties [48, 49], which may increase feed intake and thereby contribute to the observed growth enhancement in crayfish. Similar growth-promoting effects have been reported in sea cucumber [16], sea urchin [50], abalone [51], and shrimp [17]. However, higher levels of *L. japonica* extracts in feed can have negative effects on animals, such as reduced growth [23], inflammation [18], and microbiological disturbances [52]. Therefore, it is crucial to investigate the optimal dosage of *L. japonica* extract for promoting growth in different aquatic species. In the present study, it was observed that *L. japonica* extract levels exceeding 2000 mg/kg inhibited crayfish growth. One potential explanation is that nonstarch polysaccharides in *L. japonica*-derived ingredients may bind cholesterol and bile acids in the animal's digestive system, potentially affecting fat metabolism and utilization [23, 53], which may also be an important reason for the reduced growth of crayfish given high *L. japonica* extract diets.

Blood parameters are widely recognized as indicators to assess the health status of animals. Serum GOT is frequently measured to evaluate the extent of hepatic injury [54]. In the present study, dietary supplementation with *L. japonica* extract was found to support regular cellular morphology, including a well-arranged polygonal lumen and an increase in F cells. Concurrently, serum GOT and MDA levels got decreased, which may be attributed to the enhanced antioxidant system induced by the *L. japonica* extracts. Similar findings have been reported in hybrid snakehead [18], mice [55], large yellow croaker [56], and Rohu carp [20], suggesting that the *L. japonica*-derived bioactive compounds play a role in these effects. Hepatic oxidative stress, often induced by environmental perturbations or suboptimal dietary formulations [6, 7], reflects compromised antioxidant defense mechanisms primarily through lipid peroxidation processes. The detrimental impact of reactive oxygen species on nutrient bioavailability [57] highlights the critical need for optimizing antioxidant potential in aquatic species. To further explore the mechanisms underlying the reversal of hepatic injury in crayfish, we examined key indicators of hepatic oxidative stress. MDA and reduced GSH are key products of redox reactions in organisms and serve as indicators of cellular peroxidation levels [58]. Earlier research demonstrated that dietary supplementation with 200 mg/kg of *L. japonica* extract effectively mitigated oxidative stress induced by bacterial infections [20]. Such findings support the fact that significant increases could be observed in the activities of hepatic antioxidant enzymes following dietary supplementation with *L. japonica*, such as CAT and GPx, as well as a reduction in lipid peroxidation levels in the blood. This aligns with the results of the present study, with the process primarily regulated by the Nrf2 pathway [18, 19].

Earlier studies have shown that animals fed diets rich in *L. japonica* experienced reduced antioxidant capacity and compromised immune function [18, 20, 56]. According to an earlier study [17], hepatic damage may reflect alterations in the hepatopancreas immune response, often triggered by inflammation due to an overactive immune reaction. It is well established that crayfish, as crustaceans, primarily depend on innate immunity to defend against external threats [32, 38]. ACP, a crucial enzyme, plays a key role in hydrolyzing phosphate monoesters and transferring phosphate groups [59]. Compared to the control group, dietary supplementation with *L. japonica* extract significantly enhanced the hemolymph activities of ACP and LZM, key enzymes indicative of a strengthened innate immune response. Therefore, inflammation-related factors were examined to gain further insight into the connection between hepatopancreas damage and inflammation, as documented in previous studies [60]. Interestingly, the expression level of *nfkb* in the hepatopancreas decreased in crayfish fed *L. japonica*-supplemented diets, suggesting that higher doses of *L. japonica* extract may help reduce hepatic inflammation. In contrast, a diet supplemented with 2000 mg/kg of *L. japonica* extract led to an increase in hepatic *nfkb* expression. This implies that beyond an optimal level, the high load of dietary nonstarch polysaccharides derived from *L. japonica* extract may disrupt gut microbiota equilibrium or mucin balance, potentially leading to immune activation and diminished growth performance [18, 23]. Furthermore, the reduced expression levels of *alf* and *tlr* in the hepatopancreas further indicated that the inflammation observed could be linked to the presence of pathogens or their metabolites [61, 62], emphasizing the possible role of microorganisms in the inflammatory process.

Microorganisms are integral to host physiology, and there exists a highly synergistic evolutionary connection between gut microbial profiles and the crayfish immune system [63, 64]. As such, this study explores the impact of dietary *L. japonica* extract on the gut bacterial communities of crayfish, providing insights into the microbial remodeling process. The functional significance of microbial diversity, encompassing both genotypic composition and population dynamics, warrants thorough consideration [30]. Our findings demonstrate that CK diets induced intestinal dysbiosis, accompanied by compromised immune function and structural modifications. While existing literature has documented microbiota changes in response to dietary *L. japonica* supplementation [17, 27, 47, 52], the underlying mechanisms remain poorly characterized. The current investigation offers novel insights through a comprehensive comparative analysis between control and supplemented groups. Distinct clustering patterns were observed, with control groups showing significant separation from high-supplementation cohorts. Enhanced microbial richness in the *L. japonica*-supplemented groups indicates potential colonization by novel taxa, suggesting that *L. japonica* extract may mitigate CK diet-induced dysbiosis through selective microbial enrichment. Our results indicate that Firmicutes, Bacteroidota, and Proteobacteria were the primary phyla contributing to these microbial alterations, with genera such as *Citrobacter* and *Vibrio* (assigned to Proteobacteria), *Anaerorhabdus* and *Tyzzerella* (classified into Firmicutes), and *Dysgonomonas* (categorized as Bacteroidota) presenting notable changes. These findings offer potential regulatory regimes that could be essential for maintaining host health subjected to high *L. japonica* extract diets [27, 28, 34]. Notably, *Citrobacter* and *Vibrio* decreased in the gut bacterial profiles of the *L. japonica* extract-supplemented groups, together with the decrease in *Tyzzerella*. *Citrobacter* and *Vibrio*, commonly recognized as opportunistic pathogens [65–67], may explain the enhanced immune responses observed in crayfish supplemented with *L. japonica* extract. Additionally, *Tyzzerella*, potentially beneficial genera, likely contributes to polysaccharide metabolism [68, 69], supporting the growth-promoting effects of *L. japonica* extract supplementation. Furthermore, it was reported that *Tyzzerella* possesses detoxification capacity [70], explaining the increased antioxidant capacity of crayfish given *L. japonica* extract.

Key microorganisms play a crucial role in determining the functional taxonomic variation observed in gut microbiota profiles [71]. The differences in gut bacterial profiles between the *L. japonica* extract-supplemented groups and the CK group were further highlighted by the identification of several differential microbes in the intestines of crayfish fed *L. japonica*-supplemented diets. Supplementation with *L. japonica* extract resulted in the restoration of 349 unique OTUs, predominantly from Proteobacteria and Firmicutes phyla, compared to the CK group. This observation aligns with the understanding that Proteobacteria are often associated with lipopolysaccharide production, which can compromise host health by damaging the mucosal barrier [62]. The reduction in pathogenic genera *Vibrio* and enrichment of beneficial taxa *Bacillus* correlate with improved immune markers (ACP and LZM), suggesting a direct mechanistic role of microbiota remodeling in host health. These results underscore the need to elucidate the mechanisms underlying microbiota-mediated health benefits. To this end, we implemented computational analysis of health parameters and microbial profiles, generating one-dimensional feature vectors for comprehensive evaluation. Further, we identified four key microorganisms that appear to drive changes in crayfish health: *Bacillus*, *Chryseobacterium*, *Prevotella*, *Nautella*, and an unclassified genus within Proteobacteria. Among these, *Bacillus* [72, 73], *Chryseobacterium* [74], and *Prevotella* [75] are well-known genera associated with protective effects against viral infections in crustaceans, yet all of which exhibited a positive response to dietary *L. japonica* extract inclusion levels, presented the highest abundance in the LJP1.5 group, directly causing core flora dysbiosis [76].

The neutral-based hypothesis is a fundamental framework for understanding the assembly patterns of microbial communities [77]. As demonstrated by Sloan et al. [78], the limited fit of intestinal microbiota to neutral models across treatment groups indicates the predominance of deterministic processes in microbial community assembly. Within the relatively closed ecosystem of the crayfish gastrointestinal tract, bacterial populations engage in specific interactions that drive deterministic community structuring [79]. These findings emphasize the critical role of interspecies relationships, where microbial interactions outweigh abiotic factors in shaping community dynamics. In this context, niche-based theory, alongside neutral community theory, provides a critical framework for understanding microbial community assembly, emphasizing deterministic processes. From an ecological perspective, modular hubs within microbial communities may serve dual roles, functioning as generalists while potentially exhibiting greater metabolic heterogeneity and efficiency than specialists [80]. Our findings revealed elevated modular connectivity in control groups, suggesting that CK-based diets may intensify interspecies interactions within microbial networks. This pattern indicates enhanced ecological resilience, including improved nutrient utilization efficiency [81, 82] and adaptive capacity in complex microbial environments [77]. Notably, the LJP1.5 group displayed a marked predominance of specialist taxa compared to other supplementation levels. Consequently, systematic evaluation of dose-dependent effects on gut microbiota stability in crayfish warrants further investigation.

Microorganisms exhibit complex social dynamics, characterized by diverse ecological interactions including co-operative and competitive relationships [36, 80]. These interactions are often facilitated by ecological networks, which play a critical role in maintaining the stability of microbial communities [83]. Network mining revealed significantly greater complexity in microbial interactions within the LJP1.5 group, characterized by maximal node connectivity and interspecies associations. The modular architecture, representing network compartmentalization into distinct functional units, serves as a fundamental characteristic of ecological network organization [71]. In the high *L. japonica* extract-supplemented groups, four modular clusters and numerous novel modules were identified within the bacterial co-occurrence patterns, based on differential analysis across module pairs. These findings suggest that *L. japonica* extract supplementation promotes the establishment of novel ecological functions and enhances the robustness of the microbial community. Furthermore, a well-structured ecological network composed of interspecies interactions is vital for sustaining the stability and resilience of complex microecological associations [36]. In general, stable microbial communities function as negative feedback systems, where microorganisms primarily engage in competitive interactions (mathematically represented as negative correlations) [80]. These findings suggest that *L. japonica* extract supplementation may enhance microbial network stability by facilitating competitive interactions, thereby promoting ecosystem resilience and structural integrity.

Despite the valuable insights gained, this study has some limitations. The activity of the phenoloxidase (PO) system, a pivotal component of crustacean cellular immunity, was not assessed. While we documented significant enhancements in humoral immune parameters (e.g., LZM and ACP), future investigations should include measurements of PO activity and conduct in vivo challenge tests with pathogenic bacteria or viruses to fully evaluate the comprehensive immunostimulatory effects of *L. japonica* extract.

## 5. Conclusion

Taken above, dietary supplementation with *L. japonica* extracts reshaped intestinal microbiota via upregulating *Bacillus*, *Chryseobacterium*, and *Prevotella*, positively benefiting the antioxidant defense mechanisms and innate immunity of *P. clarkii*, which subsequently improved growth and hepatic histomorphology. Based on our findings, the *L. japonica* extract supplementation included in crayfish feed is recommended to be 1507.89–1614.26 mg/kg. These findings support the use of *L. japonica* extract as a sustainable feed additive to enhance crayfish health and productivity, with potential economic benefits for aquaculture.

## Figures and Tables

**Table 1:** Feed formulation (dried mass, %).

Ingredients (%)	CK	LJP0.5	LJP1.0	LJP1.5	LJP2.0
Soybean meal	25.00	25.00	25.00	25.00	25.00
Rapeseed meal	22.00	22.00	22.00	22.00	22.00
Poultry by-product meal	15.00	15.00	15.00	15.00	15.00
Wheat flour	24.00	24.00	24.00	24.00	24.00
Soybean oil	3.53	3.53	3.53	3.53	3.53
Wheat bran	5.23	5.18	5.13	5.08	5.03
α-Starch	2.00	2.00	2.00	2.00	2.00
Monocalcium phosphate	2.00	2.00	2.00	2.00	2.00
Premix^a^	1.00	1.00	1.00	1.00	1.00
Antioxidant	0.01	0.01	0.01	0.01	0.01
Anti-mildew agent	0.03	0.03	0.03	0.03	0.03
Choline chloride	0.20	0.20	0.20	0.20	0.20
*L. japonica* extract^b^	0.00	0.05	0.10	0.15	0.20
Total	100.00	100.00	100.00	100.00	100.00
Proximate nutritional analysis (%)^c^					
Crude protein	33.10	33.00	32.80	33.70	32.60
Crude lipid	6.20	6.40	6.40	6.00	6.10

^a^Premix: Contained the following per kg of the diet: Fe 170 mg; Cu 11 mg; Mn 12 mg; Zn 34 mg; Mg 60 mg; I 1.5 mg; K 200 mg; provided vitamins: VA 1700 IU; VD3 1200 IU; VE 45 mg; VK3 4 mg; VB1 4.5 mg; VB2 10 mg; VB6 15 mg; VB12 0.05 mg; niacin acid 65 mg; folic acid 5.5 mg; inositol 110 mg; D- biotin 0.1 mg; D-calcium pantothenate 23 mg. Premix was provided by Qingdao Master Biotechnology Co.

^b^ *L. japonica* extract: *L. japonica* extract was provided by Rongcheng Hongde Marine Biotechnology Co., Ltd., The proximate composition of *L. japonica* extract was presented in Supporting Information 1.

^c^Proximate nutritional analysis: The data displayed is the mean of three replicates measured.

**Table 2:** Information of primers used in this study.

Gene	Forward (5′_–_3′)	Reverse (5′_–_3′)	Amplification efficiency (%)	Source
*β-actin*	TATCCTGCGTCTGGACTTGG	CGAACGATTTCTCGCTCTGC	104.52	KR135165.1
*nfkb*	TAGTGCGTGATGATGGGTCTT	GCTGATTATGGAGGCAGAAAA	97.27	KF662471.1
*lzm*	GAGGATGTGGTCGTGGGTGA	ATTGGTCGTTCTAATGCCGC	102.92	XM_045753416.1
*alf*	CGGTTGGCGCCTCTACTACA	GCGTGCTCGATGGCTCCTG	97.82	MF185749.1
*tlr*	TTGCGTAGTGACTTGTGGAGC	CTACTGTAACGCAGGCGATGG	105.28	KP259728.1
*cat*	GCATTTGGATACTTTGAGGTG	AGAAGATGGGCGTGTTGTT	102.78	KC809959.1
*gpx*	CGAACCCTTGATGACCCTG	GAATGTCCCCAATCCTGATG	103.51	JN835259.1
*gst*	CTCCCGTCGTTGGTTACTG	GTGATCTTCACATCTCCGTCA	95.02	HQ414581.1
*mmnsod*	GCAGCCAGTGTAGGAGTAAAGG	CAAGTCCAAGCAAGGGTATCA	93.14	KC333178.1
*nrf2*	TTGAGCCAGTGTCACCCCTAA	TGCCACCGTTCCATTTCC	105.46	XM_045759458.1
*gr*	CTGGAGTACGGTTGTTGTGG	AAGGGTTGGTAGTAGGAATGG	90.22	XM_045732616.1

*Note: nrf2: NF-E2-related nuclear factor*.

Abbreviations: *β-actin*, beta-actin; *alf*, antilipopolysaccharide factor; *cat*, catalase; *gpx*, glutathione peroxidase; *gr*, glutathione reductase; *gst*, glutathione s-transferase; *lzm*, lysozyme; *mmnsod*, mitochondrial manganese superoxide dismutase; *nfkb*, nuclear factor kappa B; *tlr*, Toll-like receptor.

**Table 3:** Topological properties of microbial co-occurrence networks in *P. clarkii*-fed diets supplemented with *L. japonica* extract.

Items	Group				
	CK	LJP0.5	LJP1.0	LJP1.5	LJP2.0
Empirical network					
Similarity threshold (St)	0.93	0.93	0.93	0.93	0.93
Node number (*N*)	321	214	384	929	306
Edge number (*N*)	1889	1569	11,395	14,718	2272
Positive edges (%)	94.02	98.60	99.26	95.37	96.17
Negative edges (%)	5.98	1.40	0.74	4.63	3.83
Average degree (avgK)	11.77	14.66	59.35	31.69	14.85
Module number (*N*)	18	22	14	22	19
Average clustering coefficient (avgCC)	0.69	0.70	0.76	0.73	0.75
Modularity (*M*)	0.84	0.50	0.33	0.65	0.63
Average path distance (GD)	7.03	2.90	2.25	5.43	3.88
Random network					
Average clustering coefficient (avgCC)	0.08 ± 0.01	0.33 ± 0.00	0.54 ± 0.00	0.10 ± 0.01	0.12 ± 0.01
Modularity (M)	0.15 ± 0.01	0.06 ± 0.01	0.01 ± 0.00	0.05 ± 0.01	0.11 ± 0.01
Average path distance (GD)	2.72 ± 0.04	2.47 ± 0.01	2.05 ± 0.00	2.41 ± 0.03	2.50 ± 0.01

*Note:* Similarity threshold (St), cutoff value for edge connection based on random matrix theory. Node number (*N*), total number of operational taxonomic units (OTUs) in the network. Edge number (*N*), total number of connections (edges) between nodes. Positive edges (%), percentage of edges representing positive correlations between OTUs. Negative edges (%), percentage of edges representing negative correlations between OTUs. Average degree (avgK), average number of connections per node. Module number (*M*), number of densely connected subgroups (modules) in the network. Average clustering coefficient (avgCC), measure of the degree to which nodes tend to cluster together. Modularity (*M*), extent to which the network is subdivided into distinct modules (range: 0–1). Average path distance (GD), average shortest path length between all pairs of nodes.

**Table 4:** Growth performance.

Items	Group					ANOVA
	CK	LJP0.5	LJP1.0	LJP1.5	LJP2.0	*p*
IAW (g)	4.04 ± 0.02	4.01 ± 0.01	4.03 ± 0.02	4.01 ± 0.00	4.04 ± 0.01	0.523
FAW (g)	9.99 ± 0.15^b^	9.97 ± 0.15^b^	10.43 ± 0.09^ab^	10.78 ± 0.09^a^	10.38 ± 0.12^ab^	0.004
WGR (%)	147.31 ± 3.02^b^	148.70 ± 4.42^b^	159.00 ± 3.32^ab^	168.60 ± 2.36^a^	156.88 ± 3.43^ab^	0.008
SGR (%/day)	2.16 ± 0.03^b^	2.17 ± 0.04^b^	2.27 ± 0.03^ab^	2.35 ± 0.02^a^	2.25 ± 0.03^ab^	0.008
FCR	1.68 ± 0.05^a^	1.65 ± 0.05^ab^	1.52 ± 0.04^ab^	1.46 ± 0.03^b^	1.53 ± 0.05^ab^	0.024
SR (%)	76.00 ± 2.31	79.33 ± 4.67	80.67 ± 1.33	78.67 ± 1.76	81.33 ± 2.40	0.690
HSI (%)	12.35 ± 0.43^a^	10.62 ± 0.28^b^	10.06 ± 0.35^b^	10.24 ± 0.39^b^	9.90 ± 0.20^b^	0.010
CF (g/cm^3^)	4.57 ± 0.11	4.68 ± 0.13	4.68 ± 0.09	4.69 ± 0.07	4.52 ± 0.17	0.757

*Note:* The presented data represent the mean of three replicates. Different superscript letters indicate statistical differences at a significance level of *p* < 0.05.

Abbreviations: CF, condition factor; FAW, final average weight; FCR, feed conversion rate; HSI, hepatosomatic index; IAW, initial average weight; SGR, specific growth rate; SR, survival rate; WGR, weight gain rate.

**Table 5:** Hemolymph biochemical indicators.

Items	Group					ANOVA
	CK	LJP0.5	LJP1.0	LJP1.5	LJP2.0	*p*
MDA (nmol/mL)	7.64 ± 0.15^a^	6.92 ± 0.14^a^	5.78 ± 0.22^b^	4.96 ± 0.25^b^	5.02 ± 0.25^b^	0.001
GOT (U/L)	38.27 ± 1.28^a^	35.17 ± 1.00^ab^	34.09 ± 1.02^b^	31.57 ± 2.86^b^	28.63 ± 3.49^b^	0.040
LZM (µg/mL)	6.84 ± 0.28^b^	7.07 ± 0.32^ab^	7.33 ± 0.27^ab^	8.04 ± 0.19^a^	7.39 ± 0.11^ab^	0.025
ACP (king's unit/L)	3.16 ± 0.07^c^	3.39 ± 0.09^b^	3.64 ± 0.17^ab^	3.80 ± 0.19^a^	3.33 ± 0.03^bc^	0.009

*Note:* The presented data represent the mean of three replicates. Different superscript letters indicate statistical differences at a significance level of *p* < 0.05.

Abbreviations: ACP, acid phosphatase; GOT, glutamic oxaloacetic transaminase; LZM, lysozyme; MDA, malondialdehyde.

**Table 6:** Hepatopancreas MDA content and antioxidant-related indicators.

Items	Group					ANOVA
	CK	LJP0.5	LJP1.0	LJP1.5	LJP2.0	*p*
MDA (nmol/mg)	0.91 ± 0.01^a^	0.83 ± 0.02^b^	0.75 ± 0.03^c^	0.66 ± 0.03^d^	0.68 ± 0.04^cd^	0.001
GSH (µmol/g)	41.48 ± 2.40^c^	43.18 ± 1.66^c^	46.50 ± 1.67^bc^	53.65 ± 2.53^ab^	57.66 ± 1.58^a^	0.001
SOD (U/mg)	26.01 ± 1.02^c^	30.94 ± 0.95^b^	32.73 ± 1.27^ab^	35.57 ± 1.61^ab^	36.60 ± 0.64^a^	0.001
CAT (U/mg)	11.81 ± 0.47^b^	12.18 ± 0.43^b^	14.16 ± 0.38^ab^	16.31 ± 1.07^a^	14.83 ± 0.43^a^	0.001
GPx (U/mg)	28.41 ± 1.51^b^	32.85 ± 1.20^ab^	36.64 ± 0.82^a^	37.84 ± 1.30^a^	33.90 ± 1.36^a^	0.001
GST (U/mg)	5.57 ± 0.33^c^	6.12 ± 0.17^bc^	7.51 ± 0.23^ab^	8.49 ± 0.51^a^	8.78 ± 0.46^a^	0.001
GR (U/mg)	3.50 ± 0.06^c^	3.69 ± 0.12^bc^	4.41 ± 0.38^ab^	5.22 ± 0.55^a^	5.20 ± 0.23^a^	0.009

*Note:* The presented data represent the mean of three replicates. Different superscript letters indicate statistical differences at a significance level of *p* < 0.05.

Abbreviations: CAT, catalase; GPx, glutathione peroxidase; GR, glutathione reductase; GSH, glutathione; GST, glutathione s-transferase; MDA, malondialdehyde; SOD, superoxide dismutase.

## Data Availability

The raw sequences have been deposited in the Sequence Read Archive (SRA) database at NCBI under the accession number PRJNA854610. All data and associated code are publicly available on the open-source website GitHub (https://github.com/mineraltsai) with permission granted by the authors upon reasonable request.

## Supporting Information

Supporting Information Supporting Information 1: Proximate composition analysis of seaweed polysaccharides and related compounds, including measured values of alginic acid, fucoidan sulfate ester, mannitol, iodine, and other health indicators. Supporting Information 2: Ethical approval document from the Ethics Committee of Hunan Agricultural University (Grant Number 2024EA126) for the crayfish feed technology research project, including experimental design, animal welfare measures, and disposal protocols. Supporting Information 3: Detailed protocol for crayfish intestinal tissue fixation, dehydration, paraffin embedding, sectioning, and hematoxylin–eosin staining. Supporting Information 4: RNA extraction and cDNA synthesis protocol from crayfish intestines, including quality control steps (agarose gel electrophoresis and spectrophotometry). Supporting Information 5: Bioinformatics pipeline for microbiome data analysis (DADA2, Silva132 database annotation, MUSCLE5 alignment, and IQ-TREE phylogenetic analysis). Supporting Information 6: Statistical comparison tables (ANOSIM, Adonis, MRPP) of microbial community differences between experimental groups, based on Bray–Curtis distances.
