# Supplementary material for: Dietary Supplementation With Laminaria japonica Extract Modulates Microbial Metabolic Functions, Improving Growth Performance, Innate Immunity, and Antioxidant Capacity in Juvenile Procambarus clarkii (GIRARD, 1852)
Source: Aquac Nutr. 2025 Oct 23;2025:6896135. doi: 10.1155/anu/6896135 (PMC12575023; doi:10.1155/anu/6896135)
Supplement: Supporting Information — 1: Proximate composition analysis of seaweed polysaccharides and related compounds, including measured values of alginic acid, fucoidan sulfate ester, mannitol, iodine, and other health indicators. Supporting Information 2: Ethical approval document from the Ethics Committee of Hunan Agricultural University (Grant Number 2024EA126) for the crayfish feed technology research project, including experimental design, animal welfare measures, and disposal protocols. Supporting Information 3: Detailed protocol for crayfish intestinal tissue fixation, dehydration, paraffin embedding, sectioning, and hematoxylin–eosin staining. Supporting Information 4: RNA extraction and cDNA synthesis protocol from crayfish intestines, including quality control steps (agarose gel electrophoresis and spectrophotometry). Supporting Information 5: Bioinformatics pipeline for microbiome data analysis (DADA2, Silva132 database annotation, MUSCLE5 alignment, and IQ-TREE phylogenetic analysis). Supporting Information 6: Statistical comparison tables (ANOSIM, Adonis, MRPP) of microbial community differences between experimental groups, based on Bray–Curtis distances. [file 6896135.f1.pdf]

**Supplementary material 1**

| Item | | Standard value | Measured values |
|---|---|---|---|
| *Laminaria japonica* polysaccharides | Seaweed polysaccharides (calculated as glucose), % | ≥15 | 15.3 |
| | Alginic Acid, % | ≥20 | 28.3 |
| | Fucoidan sulfate ester, % | ≥15 | 19.5 |
| | Total, % | ≥50 | 63.1 |
| Mannitol, % | | ≥10 | 13.1 |
| Iodine | | ≥50 ppm | 53 ppm |
| Moisture, % | | ≤5 | 3.52 |
| Insoluble substances | | ≤5 | 1.05 |
| Amino acid, % | | ≥1.5 | 1.85 |
| Molecular weight (3100-13700 Daltons) | | 98.3% | |
| Appearance | | Yellow green fine particles, unique scent of kelp | |
| Health indicators | | Meet the standards for feed ingredients and additives | |

Supplementary material 2

# 湖南农业大学生物医学研究伦理委员会伦理审批件

伦审科 2024第（ 126 ）号

| 项目名称 | 小龙虾功能性饲料关键技术研发推广 | | | | |
|---|---|---|---|---|---|
| 承担专业 | 水产 | 承担责任 | ☑负责□参与 | 申请人 | 胡毅、蔡明浪 |
| 研究分类* | 1 ☑ 2 □ 3□ | 研究起止时间 | 2022.01~2026.12 | | |
| 研究类型 | 自然科学基金项目 | | | | |
| 课题来源 | ☑政府 ☑基金会 □公司 □国际组织 □其他 | | | | |

**递交审查资料：**☑项目申请书 □研究论文 □其它

**联系电话：13875910605/18351271302**

**研究主要内容：**

本项目拟规范和改良现有饲料配方及生产工艺，同时集中力量研发无绿色免疫增强剂，以提升小龙虾养殖的健康与环保水平。为迎合生产实际需要，小龙虾的生物学过程和机理需在体外研究。拟采用 1350 尾 4-10 克规格的小龙虾（性别随机）用于实验当年的采样，虾苗来源于湖南省淡水虾蟹健康养殖研究院，养殖实验均在池塘网箱（2.0 × 2.0 × 1.5 m）内进行。由于本实验涉及统计学，1350 尾小龙虾是使统计学有意义的最少动物数量。为了减少解剖和抽血带来的疼痛，我们对小龙虾进行安乐处理，使用丁香酚（1:12000）对小龙虾进行麻醉。存活的小龙虾我们将其返回湖南省淡水虾蟹健康养殖研究院。针对小龙虾尸体、组织或体液等生物材料的最终处理，我们将其袋装后冷冻，由学校实验动物中心交给有资质的公司集体回收，作无公害化处理。

**项目负责人承诺**

**我将自觉遵守实验动物福利和科学伦理原则，严格按照有关规范和标准开展实验研究，随时接受湖南农业大学生物医学研究伦理委员会的监督和检查。如违反规定，自愿接受相应处罚。**

**签字：**胡毅 蔡明浪 **日期：**

**审查结果**

所在学院审查意见：

**经审查，该研究的实验设计和方案充分考虑了安全性和公平性原则，一方面充分考虑了进行实验时的替代、减少和优化三原则，保护动物的权益，并将最大程度减轻动物的疼痛、痛苦和紧张。**

签章：湖南农业大学水产学院

日期：

伦理委员会意见：

☑同意 □修改后同意 □修改后重审 □不同意

伦理委员会主任委员签章：

伦理委员会公章：湖南农业大学生物医学研究伦理委员会

日期：

*备注：研究分类：1=动物实验； 2=动物组织或细胞实验； 3=其他

本表一式 2 份，学院签字盖章后到校科技处办理。

**Supplementary material 3**

Fresh intestines of crayfish were fixed in formalin solution for 30-50 minutes. the fixed tissue was dehydrated in 50%, 70%, 80%, 95%, and 100% alcohol for 30 minutes. after that, they were placed in a 1:1 mixture of anhydrous ethanol and xylene for 2 hours, xylene for 1.5 hours, xylene for 1.5 hours. After the clearing process. The fixed tissue was baked in a 1:1 mixture of paraffin and xylene at 40 ℃ for 40 minutes, followed by translucent waxing using paraffin I for 30 minutes and paraffin II for 40 minutes. The oven temperature was raised to 60 ℃, and the pure wax was changed three times, each time for 1-2 hours. The 10-μm paraffin sections were deparaffinized with xylene Ⅰ for 20 minutes and xylene Ⅱ for 10 minutes, and then put into various levels of alcoholic solution for 2 minutes and distilled water for 2 minutes, and then immersed in hematoxylin staining solution for 40 minutes, and then rinsed several times in distilled water until they became bluish, and then examined by microscopy. The paraffin sections that passed the microscopic examination were immersed in various levels of alcohol for 2 minutes, then in eosin solution for 20 to 30 seconds, then in 95%, pure alcohol I and pure alcohol II for 3 minutes, and finally sealed with neutral gum for microscopic observation of the tissue structure.

**Supplementary material 4**

The total RNA from the sampled intestines was isolated using Monzol Reagent Kit (Monad, Wuhan, China). The quantity and quality of the extracted RNA were assessed by agarose gel electrophoresis at 1% and spectrophotometric analysis at 260 and 280 nm. Subsequently, the total RNA was reversely transcribed into cDNA using MonScript™ RTIII All-in-One Mix with dsDNase (Monad, Wuhan, China). The following procedures was carried out: (1) 37 ℃, 2 min; (2) 55 ℃, 15 min; (3) 85 ℃, 5 min. The reaction products were stored at -80 °C.

**Supplementary material 5**

DADA2 was called for quality control, denoising, and chimera removal via the command `qiime dada2 denoise-paired` (Callahan et al., 2017). Following this, the amplicon sequence variants (OTUs) feature sequences and OTU tables were merged, and the Silva132 database was selected for annotation (Quast et al., 2012). Additionally, Muscle5 was used for multiple sequence alignment (Edgar, 2022), followed by sequence trimming using trimal (Capella-Gutiérrez et al., 2009), the construction of an evolutionary tree by maximum likelihood method using iqtree (Minh et al., 2020). Furthermore, the relative abundance of OTUs that can be observed in each sample at that sequencing depth is predicted by rarefaction, where a certain number of sequences are randomly selected from each sample separately to achieve a uniform depth.

**References**


Callahan BJ, Mcmurdie PJ, Holmes SP. Exact sequence variants should replace operational taxonomic units in marker-gene data analysis. The ISME Journal 2017;11:2639-43.

Capella-Gutiérrez S, Silla-Martínez JM, Gabaldón T. Trimal: A tool for automated alignment trimming in large-scale phylogenetic analyses. Bioinformatics 2009;25:1972-3.

Edgar RC. Muscle5: High-accuracy alignment ensembles enable unbiased assessments of sequence homology and phylogeny. Nature Communications 2022;13:6968.

Minh BQ, Schmidt HA, Chernomor O, Schrempf D, Woodhams MD, Von Haeseler A, Lanfear R. Iq-tree 2: New models and efficient methods for phylogenetic inference in the genomic era. Molecular Biology and Evolution 2020;37:1530-4.

Quast C, Pruesse E, Yilmaz P, Gerken J, Schweer T, Yarza P, Peplies J, Glöckner FO. The silva ribosomal rna gene database project: Improved data processing and web-based tools. Nucleic Acids Research 2012;41:D590-D6.

**Supplementary material 6**

| Comparison | ANOSIM | | Adonis | | MRPP | |
|---|---|---|---|---|---|---|
| | r | P | F | P | $\partial$ | P |
| Whole data | 0.277 | 0.001 | 2.606 | 0.001 | 0.547 | 0.001 |
| CK vs LJP0.5 | -0.054 | 0.801 | 0.881 | 0.647 | 0.567 | 0.202 |
| CK vs LJP1.0 | -0.004 | 0.486 | 0.92 | 0.54 | 0.515 | 0.185 |
| CK vs LJP1.5 | 0.317 | 0.012 | 2.788 | 0.008 | 0.474 | 0.005 |
| CK vs LJP2.0 | 0.337 | 0.012 | 3.481 | 0.008 | 0.446 | 0.008 |

Note: Three different permutation tests were performed (MRPP, ANOSIM and Adonis) on the basis of Bray−curtis distance. Multiple response permutation procedure (MRPP). Analysis of similarity (ANOSIM). Permutational multivariate analysis of variance (Adonis or Permanova)
